# Thank you to the *Journal of the Medical Library Association* reviewers in 2021 and 2022

**DOI:** 10.5195/jmla.2023.1701

**Published:** 2023-04-21

**Authors:** Jill T. Boruff, Michelle Kraft

**Affiliations:** 1 jill.boruff@mcgill.ca, Co-Editor in Chief, *Journal of the Medical Library Association*, Associate Librarian, Schulich Library of Physical Sciences, Life Sciences, and Engineering, McGill University, Montreal, QC, Canada.; 2 kraftm@ccf.org, Co-Editor in Chief, *Journal of the Medical Library Association*, Medical Library Director, Cleveland Clinic Foundation, Cleveland, OH, United States.

## Abstract

We sincerely thank the 214 peer reviewers in 2021 and the 171 peer reviewers in 2022 who helped evaluate and improve the quality of work published in the *Journal of the Medical Library Association* (*JMLA*).

## PEER REVIEWERS WHO SERVED IN 2021

Nancy Adams

Kendra Albright

Kristine M. Alpi

Kate Anderson

Christine Andresen

Pamela Andrews

Ebele N. Anyaoku

Catherine Arnott Smith

Marie T. Ascher

Bert Avau

Lynda Ayiku

Paul Bain

Ariana Baker

Caitlin J. Bakker, AHIP

Michael Eliot Bales

Steve Baskauf

Robert D. Beckett

Naomi Bishop

Lindsay Blake

Catherine Boden

Roxanne Bogucka

Jill Boruff, AHIP

Joshua Borycz

Marci Brandenburg

Ellen Brassil

Emily Brennan

Alison Brettle

Riley James Brian

Heather L. Brown

Roy Eugene Brown, AHIP

Christopher Sean Burns

Laureen Cantwell

Nicole Capdarest-Arest, AHIP

Tallie Casucci

Thane Chambers

Liza Chan

Deborah H. Charbonneau

Amy Chatfield

Katherine Chew

Marina Chilov

Frances Chu

Natalie Clairoux

Heather Coates

Jamie Conklin

Nicole Contaxis

Marisa Conte

John W. Cyrus

Raechel Anne Damarell

Hridi Das

Jennifer Davis

Lydia Dawe

Sandy De Groote, AHIP

Ariel Deardorff

Liz Dennett

Robin Desmeules

Patricia Devine

Nadine Dexter

Vicky Duncan

Martha Earl

Erin RB Eldermire

Nina Exner

Amy Faltinek

Rouhi Fazelzad

Mary Gracy Flaherty

Elizabeth Frakes

Melissa Christine Funaro

Kenny Garcia

Julie Glanville

Abigail Goben

Sally Gore

Kelsey Grabeel

Vera Granikov

Alyssa Grimshaw

Catherine Tess Grynoch

Jean Gudenas

Karen Elizabeth Gutzman

Rosie Hanneke

Gale Hannigan

Melissa Helwig

Margaret Henderson, AHIP

Ivan Herrera-Peco

Elizabeth Hinton

Toni Hoberecht

Lilian Hoffecker

Carmen Howard

Matt Hoy

Carrie Iwema

Timothy Johnson

Emily M. Johnson-Barlow

Doug Joubert

Ellen Justice

Kellie Kaneshiro

Julia Kelly

Jessica Kilham

Michele Klein Fedyshin

Mary L. Klem

Rachel Amelia Santose Koenig

Peter Kokol

Laura Koppen

Iris Kovar-Gough

Michelle Kraft, AHIP

Chana Kraus-Friedberg

Evan Kuehn

Erica Lake

Mariana Lapidus

Deborah L. Lauseng

Janna C. Lawrence, AHIP

Gregory Laynor

Mê-Linh Lê

Carl Leak

Norice Lee

Javier Leiva

Noah Lenstra

Paul Levay

Danica Lewis

Danielle Linden

Anne M. Linton, AHIP

Diane L. Lorenzetti

Irene Lubker

Lauren Maggio

Tara Malone

Heather J. Martin

Sarah McClung

Sean M McConachie

Karen McElfresh, AHIP

Bethany Sheriese McGowan

Lisa McGuire

Melanie McGurr

Sarah Meyer

Misa Mi, AHIP

Jolene M. Miller

Heather K. Moberly

Richard Moniz

Elizabeth Moreton

Anna Beth Morgan

Jane Morgan-Daniel

Rebecca Morin

Whitney Mortensen

Joanne Marie Muellenbach

Kavita S. Mundle

Christine Neilson

Joey Nicholson

Tyler Nix

David Nolfi

Hannah F. Norton

Ruby Nugent

Kelly K. O'Brien

Robin O'Hanlon

Rob O'Reilly

Eugenia Opuda

Ani Orchanian-Cheff

Onur Ozturk

Matthew Page

Lisa Palmer

Robin MN Parker

Cathy Pepper

Gerald Perry

Jonna Peterson

Kathleen Elizabeth Phillips

T. Scott Plutchak, AHIP, FMLA

Ariel Pomputius

Biliamin Oladele Popoola

Kimberly R. Powell

Livia Puljak

Rebecca Raszewski

Erin Reardon

Robyn Reed

Melissa L. Rethlefsen

Rebecca Reznik-Zellen

Morwenna Rogers

Elizabeth Roth

Stephanie Roth, AHIP

José Antonio Salvador-Oliván

Margaret Sampson

Hannah Schilperoort

Jan W. Schoones

Carolyn Schubert

Stephanie J. Schulte

April J. Schweikhard

Jung Mi Scoulas

Amanda Scull

Tracy C. Shields

Jean Shipman

Lindsey Sikora

Mary Simons

Judy Smith

Melanie Sorsby

Edwin Vincent Sperr, Jr.

Elizabeth Stellrecht

Gregg A. Stevens

Alice Stokes

Alisa Surkis

Stephanie M. Swanberg, AHIP

Joe Swanson

Natalie Tagge

Anneliese Taylor

Nedelina Tchangalova

Nicole Theis-Mahon

Joanna Thielen

Stephanie Tomlinson

Elizabeth Kay Tompkins

Angela Tucker

Rose Turner

Emily Vardell

Rachel Lane Walden

Philip Walker

Roland Bernard Welmaker

Monique Wessels

Gloria Willson

Alexandria Quesenberry Wilson

Lin Wu

Zhiyun Xie

Lauren M. Young

Carolyn Ziegler

## PEER REVIEWERS WHO SERVED IN 2022

Karen S. Alcorn

Alice Anderson

Christine Andresen

Katelyn Angell

Catherine Arnott Smith

Toluwase Victor Asubiaro

Paul Bain

Bridget Barry Thias

Kelsa Bartley

Skye Bickett

Lindsay Blake

Catherine Boden

Roxanne Bogucka

Joshua Borycz

Roy Eugene Brown, AHIP

Amelia Brunskill

Christopher Sean Burns

Laureen Cantwell

Robin E. Champieux

Liza Chan

Deborah H. Charbonneau

Amy Chatfield

Katherine Chew

Marina Chilov

Frances Chu

Jamie Conklin

Marisa Conte

John W. Cyrus

Jennifer Davis

Basia Delawska-Elliott

Liz Dennett

Robin Desmeules

Patricia Devine

Kerry Dhakal

Mario Enrique Diaz Barrera

Martha Earl

Erin RB Eldermire

Jonathan Eldredge

Nina Exner

Mary Gracy Flaherty

David Flynn

Elizabeth Frakes

Francesca Frati

Jacqueline Freeman

Rachel Gammons

Kenny Garcia

Kayce Gill

Shalu Gillum

Dean Giustini

Julie Glanville

Adelia Bush Grabowsky

Alyssa Grimshaw

Jean Gudenas

Rosie Hanneke

Eileen G. Harrington

Melissa Helwig

Margaret Henderson, AHIP

Marisol Hernandez

Ivan Herrera-Peco

Lilian Hoffecker

Jennifer Horton

Sara Howard

Matt Hoy

Shanda L. Hunt

Meghan Hupe

Matthew Johnson

Emily M Johnson-Barlow

Emily P. Jones

Soohyung Joo

Kellie Kaneshiro

Samantha Jan Kaplan

Susan Keller

Michele Klein Fedyshin

Mary L. Klem

Lorie A. Kloda

Helge Knüttel

Laura Koppen

Iris Kovar-Gough

Chana Kraus-Friedberg

Evan Kuehn

Elizabeth Laera

Leena Lalwani

Mariana Lapidus

Janna C. Lawrence, AHIP

Gregory Laynor

Mê-Linh Lê

Paul Levay

Paul Levay

Danielle Linden

Janice Linton

Anne M. Linton, AHIP

Elizabeth R. Lorbeer

Brady Lund

Tara Malone

Heather J. Martin

Jonah McAllister-Erickson

Sarah McClung

Daniel McDonald

Karen McElfresh, AHIP

Melanie McGurr

Sandra McKeown

Misa Mi, AHIP

Jolene M. Miller

Aida Miro-Herrans

Diana Mizrachi

Heather K. Moberly

Richard Moniz

Elizabeth Moreton

Jane Morgan-Daniel

Rebecca Morin

Martin Morris

Tyler Moses

Christine Neilson

Annie Nickum

John Novak

Rob O'Reilly

Eugenia Opuda

Ani Orchanian-Cheff

Robin MN Parker

Donald Stanley Pearson

Donald Stanley Pearson

Jessica Petrey

Brandon Pieczko

Ariel Pomputius

Kimberly R. Powell

Rebecca Raszewski

Robyn Reed

Jason B. Reed

Rebecca Reznik-Zellen

Morwenna Rogers

Guillermo Armando Ronda-Pupo

Amanda Ross-White

Stephanie Roth, AHIP

Sonia Santana Arroyo

Cynthia Marie Schmidt

Jan W. Schoones

Stephanie J. Schulte

Jung Mi Scoulas

Amanda Scull

Claire Sharifi

Pamela Shaw

Tracy C. Shields

Kay Smith

Daniela Solomon

Melanie Sorsby

Edwin Vincent Sperr, Jr.

Elizabeth Stellrecht

Gregg A. Stevens

Elizabeth Suelzer

Alisa Surkis

Stephanie M. Swanberg, AHIP

Natalie Tagge

Jill M. Tarabula

Anneliese Taylor

Michele Tennant

Nicole Theis-Mahon

Joanna Thielen

Holly Thompson

Therese Triumph

Angela Tucker

Ana Ugaz

Saeideh Valizadeh-Haghi

Kathryn Vanderboll

Kathryn Vela

Sarah May Visintini

Megan Von Isenburg

Aidy Weeks

Roland Bernard Welmaker

Alexandria Quesenberry Wilson

Lauren M. Young

Carolyn Ziegler

